# Does Risk Awareness of COVID-19 Affect Visits to National Parks? Analyzing the Tourist Decision-Making Process Using the Theory of Planned Behavior

**DOI:** 10.3390/ijerph18105081

**Published:** 2021-05-11

**Authors:** Bo-Hyun Seong, Chang-Yu Hong

**Affiliations:** 1 Chungbuk Research Institute, Cheongju 28517, Korea; sbh@cri.re.kr; 2 Division of Global & Interdisciplinary Studies, Pukyong National University, Busan 48513, Korea

**Keywords:** COVID-19, risk perception, risk reduction behavior, extended theory of planned behavior

## Abstract

This study aimed to determine whether risk awareness of coronavirus disease (COVID-19) affects visits to national parks. We analyzed the tourist decision-making process during the current pandemic using the theory of planned behavior as a framework, adding variables relevant to the pandemic, such as risk perception and risk reduction behavior, to the model. Based on a literature review, we developed a research model describing the impact relationship between risk perception, the theory of planned behavior, and risk reduction behavior and tested nine hypotheses. Results of a survey of 555 visitors to two national parks supported eight of the nine hypotheses. Although the results are limited, they reaffirm the usefulness of the theory of planned behavior in explaining tourism behavior. This work is significant in that we would be able to extend the scope of subsequent research beyond a discussion of the direct effects on optimistic perceptions (bias) and risk reduction behavior as well as visit intention, by explaining the probability even in unprecedented crises such as COVID-19. Humans may be negotiating the constraints (COVID-19) or embodied tourism need through the personal bias. Furthermore, we discuss the theoretical implications of the results for tourism behavior research.

## 1. Introduction

The world continues to experience shock over the coronavirus disease (COVID-19) pandemic. In the past, serious infectious disease situations were resolved within 5 months, but this time is different. This pandemic is different from those we have experienced in the past in that even though vaccinations have begun, and it is difficult to guarantee a complete end in 2020, even by 2021. The damage to the tourism industry is having an unprecedented impact on both the inbound and outbound markets of most countries worldwide.

When facing a major crisis, resilience, the essential power source of the mind that transforms the struggle with the crisis into happiness, surges [1]. Therefore, even if psychological contraction is caused by infectious diseases and terrorism, the speed of recovery to escape is quickly unleashed in the pattern of tourism needs. In most countries, the number of visitors is clearly increasing, especially in places located around the city, among nature-friendly spaces such as national parks, which are considered safe because of the short travel distance, depending on the intensity of COVID-19 countermeasures. Although social concerns due to the outbreak of infectious diseases are emerging as travel avoidance, it is time for an academic understanding of the fact that exploration activities in natural spaces are continuously being carried out.

Some researchers have suggested that pandemic-level epidemic cycles may occur more frequently in the near future [2,3]. It can be assumed that participation in outdoor recreation to relieve tourism needs and fight infectious diseases will continue. In terms of effective management of outdoor recreation spaces such as national parks, there is a need to examine the psychological factors related to the decision to carry out exploration activities.

The usefulness of the theory of planned behavior as a theoretical framework for explaining the process of various human actions, including tourism, has been widely recognized [4,5,6,7,8,9,10,11,12,13]. The planned behavior theory presents attitudes, subjective norms, perceived sense of behavioral control, etc., as leading variables that determine behavioral intentions, and explains the decision-making process in which specific actions are performed through influencing relationships between the variables. Intention is the most direct and immediate precursor to predicting behavior [14]. Recently, many studies have attempted to expand the theory by adding new factors to those in the original model (attitudes, subjective norms, and perceived behavioral controls) to enhance the model’s ability to elucidate the process of tourism behavior, in order to contribute to a greater understanding of the influential factors [15,16,17,18,19,20,21,22].

This study aimed to determine whether risk awareness of COVID-19 affects visits to national parks. We analyzed the tourist decision-making process during the current pandemic using the theory of planned behavior as a framework, adding variables relevant to the pandemic, such as risk perception and risk reduction behavior, to the model. Considering that people continue to visit natural tourist attractions such as national parks, we conducted a survey of visitors to verify the correlations between COVID-19 risk perceptions, risk reduction behaviors, and the theory of planned behavior. Existing studies [23,24,25,26,27] suggest that the higher the level of risk perceived by tourists, the more likely they are to engage in risk-reducing behaviors, warranting this study’s examination of COVID-19 risk perceptions and risk-reduction behaviors as additional variables expanding the theory of planned behavior.

Under the special circumstances of the COVID-19 pandemic, we judge that the academic discussion on the influencing relationship of each variable can be more meaningful. Primarily, having the opportunity of this research work is meaningful and crucial because it would be hard for Korean researchers to find related research cases. In order to achieve the purpose of the study, we established hypotheses based on the analysis of tourism behaviors such as an expanded theory of planned behavior, risk perception, and risk reduction behavior, and reviewed the hypotheses based on the results of a survey using structural equation modeling. The findings have important theoretical implications for tourism behavior research, and also expatiates practical strategies for managing nature-friendly tourist spaces such as national parks.

## 2. Conceptual Note

### 2.1. Risk Perception and COVID-19

Academic interests in the risk construct has existed since one researcher [28] suggested risk was a critical component of economic activity in the 1940s. Since its inception, the concept of risk has been examined across a range of disciplines, including geology [29], sociology [30], psychology [31], marketing [24,32] and tourism [33,34,35,36].

Ordinary people have an intuitive sense of risk, which is referred to as “risk perception” [37]. Risk perception is not considered to be a form of objective and stochastic perception, but a subjective perception in selective situations [24]. Within the field of tourism studies, scholars have approached the study of risk from various perspectives such as perceived risks, destination safety, social construction of risk and tourists’ narratives, tourists’ worries, and risk-taking [35].

In marketing and similar or related areas, risk perception is defined as a person’s level of uncertainty and perception of adverse consequences of buying a product or service [38]. In tourism studies, risk perception refers to the anxiety that tourists perceive and experience while purchasing and consuming tourism services [39]. Recognizing high risks in the process of exploring related information before visiting tourist attractions will limit decisions due to worries and fears, and affect satisfaction and loyalty to tourist products and attractions [40,41,42].

One study [43] comprehensively explored risk perception in the context of travel and tourism and found seven dimensions of this area—equipment, financial, physical, psychological, satisfaction, social, and time. Later, another study [40] added to this work with the addition of health, terrorism, and political instability. Seven categories of political instability have been considered in the field of perceived risk—terrorism, unfamiliar food, cultural barriers, national, political, religious doctrines, and misdemeanors [44]. Later work [45] classified political risks, such as terrorism, political instability, and war/military conflict, environmental risks, such as natural disasters, landslides, difficult access to hospitals, life-threatening diseases, lack of clean food and water, and planned risks due to unreliable aviation and inexperienced operators.

Many studies have explored perceived risks specific to nature destinations and travel and have found new dimensions of this type of risk [33,46,47,48,49]. There is no doubt that from 2020 to the present, the most recognized risk in most areas, including tourism, has been COVID-19, and this risk is a constraint on participation in tourism activities. Infectious diseases have been shown to be an important factor in many tourism-related risk perception studies [33,35,44,45,50]. In Korea, the number of visitors to major tourist sites in 2020 decreased by 40% to 60% from January to December.

In contrast, the decrease in visitors to national parks was considerably less than that of general tourist attractions. According to the National Park Management Corporation [51], the number of visitors to national parks decreased by 19.2% in 2020 compared with the same period in 2019 (19,899,596 → 16,081,996), which is drawing keen attention. We understand that, after COVID-19 fallout, there is a risk reduction behavior in which pent-up desires to engage in tourism are replaced with desires for activity in outdoor recreational spaces that are deemed relatively safe. This is because tourists tend to minimize the uncertainty that can arise from the purchase of tourism products if the perceived risk level is low [50,52,53].

### 2.2. Risk Reduction Bahavior

Manning [54] explained through a congestion model that tourists who are aware of the occurrence of congestion due to excessive demand for tourist attractions may engage in site displacement behavior. As a result, congestion complaints shift to less congested areas, or tourists engage in “confusion avoidance,” which means giving up on the activity itself [55]. Other studies [56,57] verified tourists’ coping responses, such as navigating and moving toward a less congested space, and changes in visit time.

The coping behavior described in the congestion model provides meaningful implications for outdoor recreation spaces such as national parks, where many visitors visit, even in the aftermath of COVID-19, because tourists modify their behavior [23] by refraining from tourism activities or avoiding crowded places to reduce perceived risks. In addition, further research is needed to determine the causes of risk reduction behaviors, such as how consumers respond when they are at risk [58].

The risk reduction behavior of visitors to national parks examined in the present study can be defined as efforts to cope with reducing the risk of COVID-19 infection on trails. The main purpose of social distancing prevention measures has been to reduce congestion or minimize face-to-face contact in certain spaces by quarantine level (level 1, level 1.5, level 2, and level 3 in Korea). These measures are carried out along with those on the mandatory wearing of masks. In accordance with these quarantine guidelines, it would be possible for visitors to respond in the course of exploration activities by determining their own guidelines for behaviors [59] to minimize risk.

As a tourist or consumer, when risk-taking is present, an individual comes up with his or her own countermeasures to reduce the risk [24,25,26], which can be described as a risk-reduction strategy. In other words, strategies to minimize adverse consequences and reduce the risk that may arise in the purchasing process can help attenuate the uncertainty or dissatisfaction that customers themselves seek [60,61].

In the field of travel and tourism, some risk reduction strategies have also been reported in the research. One study [25] explored risk perceptions toward smartphone usage and consequent risk reduction strategies through a backpacker’s survey. Another study on backpackers [27] found that tourists sought information in stores and on the Internet, and from travel agents to relieve their sense of risk. Likewise, one author [23] found that traveling in the company of friends, avoiding crowded areas, and using local tour guides were all important risk relievers for backpackers abroad.

Risk can be considered a limiting factor for tourism and leisure activities. Risk reduction behavior is sometimes considered a strategic action similar to leisure constraint negation, which is widely discussed in the literature on tourism and leisure. Some researchers [62] have criticized the existing leisure constraints model, supporting the view that leisure participation is not through complete absence but through active negotiation among constraint factors. Based on this argument, in contexts similar to risk reduction behavior or risk reduction strategies, constraint negotiation studies have explained that negotiation strategies directly and indirectly reduce the impact of constraint factors [63,64,65,66,67,68].

### 2.3. Extended Theory of Planned Behavior

The theory of planned behavior [69] is an extended version of Fishbein and Ajzen’s theory of reasoned action. It has been widely used in a variety of psychological behavioral contexts to describe the decision-making process in which individuals perform specific behaviors [21,70]. It is considered the dominant theory for explaining behavior in a wide range of fields, such as health behavior, learning behavior, consumer behavior, environmentally friendly behavior, and tourism [5,9,10,11,12,13].

According to the theory of planned behavior, attitudes, subjective norms, and perceived behavioral controls act as determinants of behavioral intention, which, in turn, influences behavior [33]. In brief, the theory posits that the key to explaining behavior is intention. Intention is the most direct and immediate antecedent of overt behavior. Intention is the subjective probability that an individual will engage in a given behavior [14].

However, the low accountability of the behavior of the three independent variables—attitudes, subjective norms, and perceived behavioral controls—of the theory of planned behavior has been the basis for expanding the theory to strengthen the explanatory power of human behavior [33,71]. As a result, the expanded theory of planned behavior emerged. Many tourism studies have employed the expanded theory of planned behavior by incorporating additional variables [15,16,17,18,19,20,21,22].

For example, behavioral intentions in a certain context could be well predicted with theory of planned behavior constructs and additional variables, such as “visa exemption” [17], “environment related variables” [16], “motivation” [18], “perceived risk and uncertainty” [21], “risk perception” [33], “winescape (winery tour)” [22], “desires” and “anticipated emotions” [72], and “destination image and travel constraints” [20].

Prior studies that have expanded the theory of planned behavior to explain the tourist decision-making process [24,26,73] have shown a positive (+) causal relationship between risk perception and risk reduction behavior. Others have conducted experiments on the process of risk reduction behavior using psychological variables such as attitudes formed by risk [21,33,74,75]. The above studies suggest that COVID-19 risk perception and coping behavior are important variables for expansion of the theory of planned behavior. 

## 3. Research Model, Hypotheses and Methodology

### 3.1. Research Model and Hypotheses

**Figure 1:**
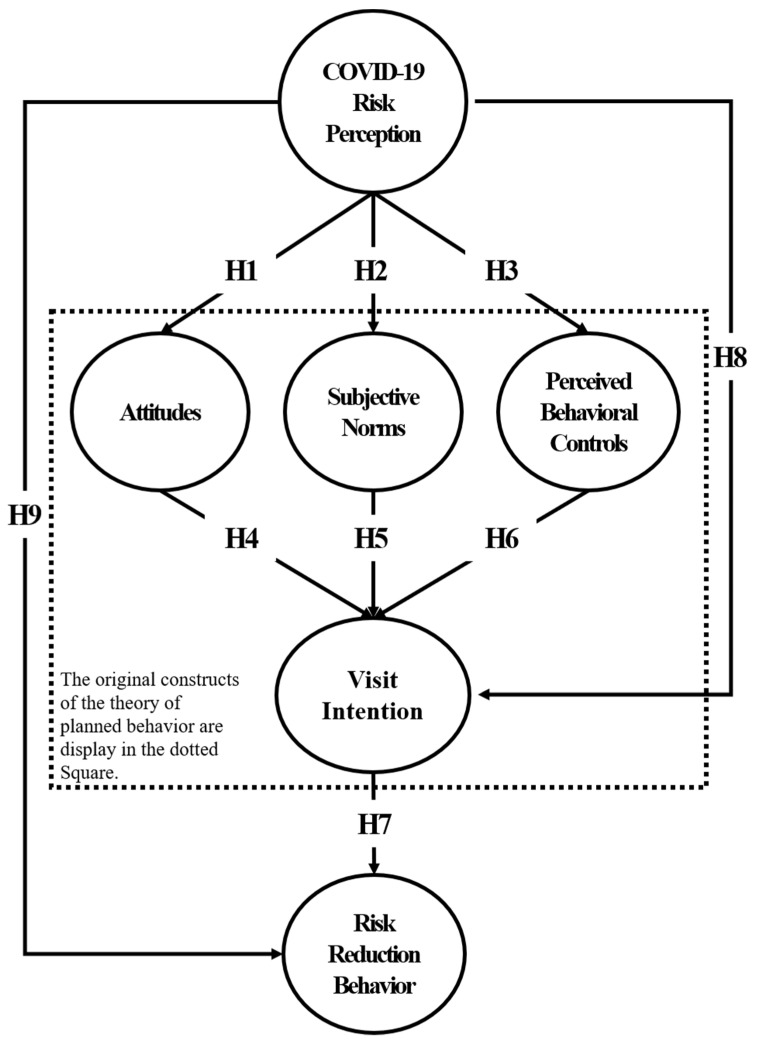
Research model.

Based on the literature study, we developed a model describing the intention of continuous visits by visitors to national parks (see Figure 1) and developed nine related hypotheses. Hypotheses 1 through 3 explain how COVID-19 risk perception affects attitudes, subjective norms, and perceived behavioral controls. The research model and hypothesis of our study were based on existing findings [21,33,74,75] describing the significant relationships between risk perception and attitudes, subjective norms, and perceived behavioral controls.

**Hypothesis 1.**  *Risk perception of COVID-19 will have a negative effect on attitudes.*

**Hypothesis 2.**  *Risk perception of COVID-19 will have a negative effect on subjective norms.*

**Hypothesis 3.**  *Risk perception of COVID-19 will have a negative effect on perceived behavioral controls.*

Hypotheses 4–6 explain how attitudes, subjective norms, and perceived behavioral controls affect the degree of continuous visits, and the results of the previously discussed studies on the theory of planned behavior [5,10,13,69]. Hypothesis 7 explains that the degree of persistent visits has a positive effect on coping behavior.

**Hypothesis 4.**  *Attitudes will have a positive effect on the degree of continuous visits.*

**Hypothesis 5.**  *Subjective norms will have a positive effect on the intention to visit continuously.*

**Hypothesis 6.**  *Perceived behavioral controls will have a positive effect on the degree of continuous visits.*

**Hypothesis 7.**  *The intention to continuously visit will have a positive effect on risk-reduction behavior.*

Hypothesis 8 was developed based on prior research [33,74,75] that demonstrated that COVID-19 risk perception has a negative effect on the degree of continuous visits. Hypothesis 9 was established based on prior research [23,25,27,60,61] that indicated that COVID-19 risk perception has a definitive effect on risk reduction behavior.

**Hypothesis 8.**  *COVID-19 risk perception will have a negative effect on the intention to visit continuously.*

**Hypothesis 9.**  *COVID-19 risk perception will have a definitive effect on risk-reduction behavior.*

### 3.2. Data Collection and Analytic Design

#### 3.2.1. Survey Questionnaire Design

The questionnaire consisted of 36 questions, including items to measure a total of six variables included in the hypothesis and items to collect data on the demographic characteristics of the respondents. The items were first derived based on literature research and existing related research works and were finalized after discussion and supplementation, with three tourism experts.

Five items for COVID-19 risk perception measurement were reconstructed from relevant studies [35,44,45] by extracting elements related to health threats. Three items for measuring risk reduction behavior were based on a study on congestion avoidance and risk reduction behavior [25,27,54,60,61]. Finally, measurements of attitudes, subjective norms, perceived sense of behavioral control, and intention to continuously visit were compiled by referring to prior studies [4,69,76,77].

#### 3.2.2. Survey Target and Research Methods

The survey was conducted in the parking lot at the entrance to Boriam Temple Park, located in Hallyeo Maritime National Park, and at the shelter and parking lot of Gyeranjae Park at Woraksan National Park. Boriam is the only mountain park in Hallyeo Maritime National Park, located at an altitude of 650 m. Therefore, both sites where the survey was conducted were national parks and mountain parks. In addition, since there were no restaurants concentrated in the national park area, it is recognized as a place to enjoy exploring activities while complying with COVID-19 regulations.

In cooperation with the National Park Office of Woraksan and Hallyeo Maritime, the survey was conducted for 2 days (Saturday, 24 October 2020, to Sunday, 25 October 2020), and only adult visitors were eligible to participate. Pre-trained survey investigators were dispatched to handout and collect the surveys using a convenience sampling method. After providing informed consent, participants were asked to complete the self-administered questionnaire collected at the site.

A total of 580 questionnaires were distributed and retrieved. After excluding surveys with potential problems, 555 valid samples were used for the final analysis. Data analysis was conducted using SPSS Statistics 23 and Amos 18.0 (IBM Corp., Armonk, NY, USA). The validity of the measurement variables was verified through confirmatory factor analysis, correlation analysis, and an examination of reliability (using Cronbach’s alpha). Finally, the hypotheses were verified through structural equation modeling analysis.

## 4. Results

### 4.1. Demographic Characteristics of the Respondents

A total of 555 valid questionnaires were collected to identify demographic characteristics such as gender, residence (place of address), age, occupation, household average monthly income, and educational background (see Table 1). There were no significant differences in gender (men = 50.7% and women = 49.3%), and it was found that people of all ages visited evenly. In addition, 60.6% (23.6% under 4–5 million won (USD 3600–4400) and 37.0% over 5 million won (USD 4400)) answered that households earn more than 4 million won (USD 3600) a month. Gyeongnam province (20.4%), which was one of the survey sites, showed the highest rate of residence, and relatively large percentages of people visited from nearby areas such as Jeonnam Province (8.8%) and Daegu Metropolitan City (8.2%).

### 4.2. Validity and Reliability of the Measurement Items

Results of the confirmatory factor analysis to verify the validity of the measurement items indicated that the model fit indices from the measurement model did not meet the minimal standards (χ²/df = 3.898 (χ² = 1181.201, df = 303), root mean squared residual (RMR) = 0.043, root mean square error of approximation (RMSEA) = 0.072, goodness of fit index (GFI) = 0.869, normed fit index (NFI) = 0.908, relative fit index (RFI) = 0.893, incremental fit index (IFI) = 0.930, Tucker-Lewis index (TLI) = 0.918, and comparative fit index (CFI) = 0.930)) (see Table 2). As a verifying result of convergent validity of measuring items, we removed one COVID-19 risk perception item whose standardized factor loading value (0.304) did not meet the standard (0.5–0.95).

Confirmatory factor analysis was re-implemented after deleting unmet survey items. The results showed that most of the fit indices of the measurement model fell under the category within the recommended criteria (χ²/df = 3.227 (χ² = 851.972, df = 64), RMR = 0.030, RMSEA = 0.063, GFI = 0.900, NFI = 0.932, RFI = 0.917, IFI = 0.952, TLI = 0.941, CFI = 0.952). The scores of each measurement item of construct reliability (CR) (higher than 1.965, *p* < 0.05), standardized factor loading (0.5–0.95), CR (recommended range—higher than 0.7), and average variance extracted (AVE) (recommended range—higher than 0.5) all represented more than the reference value, thus achieving convergent validity.

Discriminant validity was estimated by comparing the square root of the AVE for a given construct with the correlations between that construct and all other constructs. All square roots of the AVE were greater than the absolute correlations between pairs of constructs recommended in prior research [78], indicating that discriminant validity was supported. Table 3 shows that the diagonal elements have been replaced by the square roots of AVE ranging from 0.61 to 0.89, which were greater than their correlation coefficients off the diagonal ranging from −0.21 through 0.75. In sum, the measurement model has construct validity and reliability.

### 4.3. Hypothesis Testing

A structural equation model analysis was conducted on the entire sample (*n* = 555) to verify hypotheses 1–9 (Table 4). All fit indices, such as RMR, CFI, TLI, RMSEA, and NFI, had acceptable values, except χ²/df. Consequently, the model’s goodness of fit was determined to be acceptable.

Results of the analysis indicated that COVID-19 risk perception had a negative effect on attitudes, subjective norms, and perceived behavioral controls, supporting hypotheses 1, 2, and 3. COVID-19 risk perception is a constraint factor that negatively affects psychological variables at the individual level, such as attitudes and perceived behavioral controls with regard to visits to national parks. From these findings, it can be inferred that the number of visitors has decreased compared with 2019, although the decrease is small, compared with that of general tourist attractions.

Similar to the findings of previous studies [4,21,79,80,81,82,83], hypotheses 4, 5, and 6 were also supported, as the main parameters of the theory of planned behavior (attitudes, subjective norms, and perceived behavioral controls) were shown to have definitive effects on the intention to visit national parks. In this model, the influence of perceived behavioral control was the most apparent.

On the other hand, the direct permanence relationship between COVID-19 risk perception and the intention of continuous visits was not significant; therefore, hypothesis 8 was rejected, but the impact relationship between COVID-19 risk perception and coping behavior was significant. In particular, COVID-19 risk perception was found to have a stronger effect on risk-reduction behavior (0.546) through a decision-making process that mediates the main variables of planning behavior theory rather than the direct effect on risk-reduction behavior (0.157). In other words, it adds validity to our research model.

## 5. Discussion

This study assumed that visits to natural tourist attractions, such as national parks, continue despite awareness of the risk of COVID-19 infection. The purpose of the analysis was to expand the theory of planned behavior to determine whether tourists intend to continue their exploration activities safely by minimizing risks through less crowded trails and minimizing interpersonal contact. Based on the literature review, we developed a research model describing the impact relationship between risk perception, planning behavior theory, and risk reduction behavior, and developed and tested nine hypotheses.

The results of the survey of 555 park visitors supported eight of the nine hypotheses. Although the results are limited to visitors of national parks, they reaffirm the usefulness of the theory of planned behavior in explaining tourism behavior. The study is also meaningful in that COVID-19 risk perception was expanded beyond a discussion of its direct effects on visit intention, risk reduction behavior, etc., to an explanation of how the embodied tourism needs of humans are expressed in the unprecedented crisis of the pandemic. In particular, visits to national parks affect both the positive correlation and personal abilities of individuals as well as the compliant groups, such as attitudes, subjective norms, and perceived behavioral controls. Perceived behavioral control has the greatest impact on intention to visit, and the trail where the survey was conducted is accessible using a private vehicle. This hiking trail has low levels of congestion, making it easier to cover mountainous areas while practicing social distancing. It can be inferred that perceived behavioral control may have served as a major factor in engagement in exploration activities. 

It has been pointed out in prior studies on the theory of planned behavior that subjective norms exhibit lower explanatory power than attitudes [69,71,84,85,86,87,88]. It is worth noting that in the present study, subjective norms showed a higher level of explanation for intention to visit. Due to the nature of COVID-19, the decision attributes of the group are an important factor in participation in certain tourism activities because the infection spreads primarily through close social contact, such as with family, friends, and co-workers.

COVID-19 risk perception has a negative effect on attitudes, subjective norms, and perceived behavioral controls, which are psychological variables that directly or indirectly affect behavioral inducement and function as constraint factors. On the other hand, it was found that places with low density, such as national parks, were more likely to be selected and that risk reduction behavior was actively performed at these sites. This can also be inferred from the fact that negotiations over leisure constraints [89] are taking place, which means strategies to reduce or avoid risk. Furthermore, the leisure we enjoyed before COVID-19 involved activities in environments that did not require as many constraints. In the secured situation without a serious pandemic crisis, namely COVID-19, it was also decided and achieved through active negotiating processes among the constraint factors [90]. In other words, leisure constraints had existed in our lives as always. 

Visitors tend to choose a nature-friendly space such as a national park based on the social perception that it is safe from COVID-19. Even in determining behavior at a national park site, we should pay attention to the significance of the impact of optimistic bias [91,92], which refers to the subjective judgment that risk reduction is well-controlled by carrying out risk reduction behaviors. This is consistent with the results of a prior study [49,93], in which risk control, such as minimizing interpersonal contact, appeared to reduce the severity of health risks related to the environment in everyday life. 

## 6. Conclusions

Our study highlights the practical implications of controlling visitors to national parks and efficient management for coping with infectious diseases. As our findings show, there is a social atmosphere in which national parks feel relatively safe, and optimistic bias will possibly work on individuals with high levels of specialization (highly educated group). As this may lead to frequent exploration of national parks, it is also necessary for the government to carry out more active measures that promote social distancing in certain situations.

In contrast, we may consider the advantages of utilizing national parks. A visit to a national park is one of the few methods of relieving social stress caused by the spread of infectious diseases, such as "corona blue." Therefore, the government should consider ways to temporarily open some of the trails that have been closed, in order to meet tourists’ needs when reviewing measures to prevent social distancing. In addition, it is necessary to establish a smart technology system, such as developing an app that can provide a congestion index by trail in real time, such as a congestion management system. It is also necessary to improve the maintenance of sanitation and cleanliness of indoor facilities, such as toilets and visiting information centers.

This study had several limitations. The study could not be efficiently carried out due to social distancing measures. On-site investigations were restricted. We believe that for our next study, additional non-face-to-face investigative designs, such as online research, will be necessary. In addition, further research is needed to analyze the factors that affect the types of activities that are preferred by tourists, including not only national park visitors but also ordinary citizens.

## Figures and Tables

**Table 1:** Demographic characteristics of the respondents.

Item		*n* (%)	Item		*n* (%)
**Gender**	**Men**	287 (51.7)	Education level	Less than high school	146 (26.3)
	Women	268 (48.3)		Attending college	45 (8.1)
Age	20s	89 (16.0)		Bachelor’s degree	322 (58.0)
	30s	71 (12.8)		More than Graduate school	42 (7.6)
	40s	123 (22.2)		Seoul Metropolitan Government	38 (6.8)
	50s	165 (29.7)		Pusan Metropolitan City	19 (3.4)
	Older than 60	107 (19.3)		Daegu Metropolitan City	42 (7.6)
Household average monthlyincome	Less than 900 USD	7 (1.3)		Incheon Metropolitan City	35 (6.3)
	900–1800 USD	23 (4.2)		Gwangju Metropolitan City	7 (1.3)
	1800–2700 USD	90 (16.2)		Daejeon Metropolitan City	12 (2.2)
	2700–3600 USD	109 (19.6)		Ulsan Metropolitan City	6 (1.1)
	3600–4400 USD	135 (24.3)		Gyeonggi Province	94 (16.9)
	Over 4400 USD	191 (34.4)		Kangwon Province	45 (8.1)
Occupation	Self-employed	70 (12.6)		Chungbuk Province	84 (15.1)
	Professional	77 (13.9)		Chungnam Province	22 (4.0)
	Government officer	71 (12.8)		Jeonbuk Province	13 (2.3)
	Farmer	20 (3.6)		Jeonnam Province	15 (2.7)
	Student	35 (6.3)		Gyeongbuk Province	81 (14.6)
	Housewife	89 (16.0)		Gyeongnam Province	38 (6.8)
	Office worker	140 (25.2)		Jeju Special Self-Governing Province	1 (0.2)
	Others	53 (9.6)		Sejong Metropolitan Autonomous City	3 (0.5)

**Table 2:** Demographic characteristics of samples.

Variables and Observed Variables	Factor Loading	Variances	CR	AVE
Risk Perception of COVID-19				
The National Park Trail is also not safe from COVID-19.	0.682 ***	0.478	0.862	0.611
There is a lack of information on exploring national parks during the COVID-19 crisis.	0.723 ***	0.369		
I am concerned about prevention and hygiene issues with regard to indoor facilities such as toilets and shelters in national parks.	0.804 ***	0.327		
There is a lack of exploration programs in which to participate safely during the COVID-19 crisis.	0.794 ***	0.269		
**Attitudes**				
I like to visit the national park.	0.875 ***	0.126	0.972	0.874
I think a trip to the national park is a happy thing.	0.883 ***	0.076		
I think positively about the national park tour.	0.901 ***	0.099		
To me, a tour of the national park is worthwhile.	0.933 ***	0.076		
A tour of the national park will bring me good results.	0.890 ***	0.126		
**Subjective Norms**				
My family thinks positively about my visit to the national park.	0.761 ***	0.218	0.971	0.847
My friends think positively about my visit to the national park.	0.890 ***	0.119		
My acquaintances think positively about my visit to the national park.	0.930 ***	0.080		
My family will want me to visit the national park.	0.883 ***	0.132		
My friends will want me to explore the national park.	0.882 ***	0.137		
My acquaintances will want me to explore the national park.	0.885 ***	0.139		
**Perceived Behavioral Controls**				
I can visit the national park whenever I want.	0.836 ***	0.231	0.899	0.642
I have enough economic power to explore the national park.	0.757 ***	0.255		
I have time to explore the national park.	0.725 ***	0.378		
It is easy to learn the skills necessary to visit the national park.	0.657 ***	0.443		
I can easily find the information I need to visit the national park.	0.777 ***	0.271		
**Visit Intention**				
I will try to visit the national park from now on.	0.914 ***	0.084	0.961	0.891
I will recommend a tour of the national park to others.	0.891 ***	0.083		
I am sure that I will continue my tour of the national park	0.878 ***	0.128		
**Risk Reduction Behavior**				
I will choose a hiking trail that is expected to have fewer visitors.	0.847 ***	0.139	0.890	0.730
I will minimize the time on the trail where others are.	0.781 ***	0.225		
I will try to comply with COVID-19 regulations when visiting.	0.777 ***	0.352		

Note: *** *p* < 0.001. AVE = average variance extracted, CR = construct reliability.

**Table 3:** Summary of discriminant validities, correlations, means, and standard deviations.

Construct	RP	AT	SN	PBC	VI	RRB
RP	**0.61** ^1^					
AT	−0.21	**0.87**				
SN	−0.12	0.75	**0.85**			
PBC	−0.21	0.67	0.64	**0.64**		
VI	−0.12	0.61	0.61	0.66	**0.89**	
RRB	0.09	0.39	0.40	0.42	0.58	**0.77**
Mean	3.12	4.20	4.01	3.88	4.21	3.91
Std. Dev.	0.62	0.69	0.69	0.67	0.60	0.69

Note: ^1^ Bold numbers on the diagonal are the square root of average variance extracted; off-diagonal numbers are the correlations among constructs. AT = attitudes, PBC = perceived behavioral controls, RP = risk perception, RRB = risk reduction behavior, SN = subjective norms, VI = visit intention.

**Table 4:** Summary of the tested hypotheses.

Hypothesized Path		Path Coefficient	t	Results
H1	Risk Perception of COVID-19 → Attitudes	−0.24 ***	−4.37	Supported
H2	Risk Perception of COVID-19 → Subjective Norm	−0.16 *	−2.87	Supported
H3	Risk Perception of COVID-19 → Perceived Behavioral Control	−0.20 ***	−4.39	Supported
H4	Attitudes → Visit Intention	0.19 *	3.26	Supported
H5	Subjective Norms → Visit Intention	0.21 ***	4.04	Supported
H6	Perceived Behavioral Control → Visit Intention	0.41 ***	5.24	Supported
H7	Visit Intention → Risk Reduction Behavior	0.54 ***	8.89	Supported
H8	Risk Perception of COVID-19 → Visit Intention	0.04	0.99	Not Supported
H9	Risk Perception of COVID-19 → Risk Reduction Behavior	0.16 ***	4.06	Supported

Note: * *p* < 0.05; *** *p* < 0.001.

## Data Availability

Not applicable.
